# Novel Sigma-2 receptor ligand A011 overcomes MDR in adriamycin-resistant human breast cancer cells by modulating ABCB1 and ABCG2 transporter function

**DOI:** 10.3389/fphar.2022.952980

**Published:** 2022-08-31

**Authors:** Zhanwei Zeng, Shiyi Liao, Huan Zhou, Hongyu Liu, Jiantao Lin, Yunsheng Huang, Chenhui Zhou, Daohua Xu

**Affiliations:** ^1^ Guangdong Key Laboratory for Research and Development of Natural Drugs, School of Pharmacy, Guangdong Medical University, Zhanjiang, China; ^2^ Department of Pharmacy, Qingyuan People’s Hospital, The Sixth Affiliated Hospital of Guangzhou Medical University, Qingyuan, China; ^3^ Key Laboratory of Traditional Chinese Medicine and New Pharmacutical Development, Department of Pharmacology, Guangdong Medical University, Dongguan, China; ^4^ School of Nursing, Guangdong Medical University, Dongguan, China

**Keywords:** multidrug resistance, MCF-7/ADR, sigma-2 receptor, ABCB1, ABCG2

## Abstract

Multidrug resistance (MDR) is thought to be one of the main reasons for the failure of chemotherapy in cancers. ATP-binding cassette subfamily B member 1 (ABCB1) or P-glycoprotein (P-gp) and ATP-binding cassette subfamily G member 2 (ABCG2) play indispensable roles in cancer cell MDR. Sigma-2 (σ_2_) receptor is considered to be a cancer biomarker and a potential therapeutic target due to its high expression in various proliferative tumors. Recently, σ_2_ receptor ligands have been shown to have promising cytotoxic effects against cancer cells and to modulate the activity of P-glycoprotein (ABCB1) *in vitro* experiments, but their specific effects and mechanisms remain to be elucidated. We found that A011, a σ_2_ receptor ligand with the structure of 6,7-dimethoxy-1,2,3,4-tetrahydroisoquinoline, showed promising cytotoxicity against breast cancer MCF-7 and adriamycin-resistant MCF-7 (MCF-7/ADR), induced apoptosis, and reversed adriamycin (ADR) and paclitaxel resistance in MCF-7/ADR cells. Furthermore, we demonstrated that A011 increased the accumulation of rhodamine 123 and mitoxantrone in MCF-7/ADR cells. A011 significantly decreased the ATPase activity of the ABCB1 and down-regulated ABCG2 protein expression. In addition, A011, administered alone or in combination with ADR, significantly inhibited tumor growth in the MCF-7/ADR tumor-bearing nude mouse model. A011 may be a potential therapeutic agent for the treatment of tumor resistance.

## Introduction

Female breast cancer is one of the most common malignancies in the world. Breast cancer surpassed lung cancer to become the most prevalent cancer in 2020, with an estimated 2.3 million new cases (Sung et al., 2021). Over the past decades, tremendous progress has been made in the development of chemotherapeutic drugs. However, tumor recurrence and metastasis remain one of the major challenges in cancer treatment during the long-term course of chemotherapy (Warren, 2009). Most anti-tumor drugs inevitably show reduced drug efficacy and tumors exhibit multidrug resistance (MDR) to chemotherapeutic drugs during long-term chemotherapy, leading to tumor recurrence in patients. It has been reported that over 90% deaths in the chemotherapy population are associated with MDR (Bukowski et al., 2020). Various mechanisms have been reported to be involved in the development of MDR in tumor cells, including ATP-binding cassette (ABC) transporter family-mediated drug efflux, apoptosis down-regulation and epigenetic regulation, etc., of which the ABC transporter family is the most widely studied (Abraham et al., 2009; Rocha et al., 2018; Assaraf et al., 2019; Bukowski et al., 2020).

The ABC transporter superfamily is one of the largest families of transmembrane proteins, consisting of 49 ABC transporter members (Asif et al., 2020). Structurally, ABC transporters are characterized by a common structure: two nucleotide-binding domains (NBDs), which can bind and hydrolyze ATP, and two transmembrane domains (TMDs), which utilize the energy provided by ATP hydrolysis to transport substrates outside the cell. Functionally, most ABC transporters can transport the substrates produced during cellular metabolism (e.g., sugars, lipids, ions, peptides, amino acids and toxic components) from the cytoplasm to outside the cell membrane, and thus play an important role in maintaining normal physiological and pathological processes in the organism (Borst and Elferink, 2002; Theodoulou and Kerr, 2015). Dysregulated ABC transporters are associated with tumor resistance (Dean et al., 2001; Amawi et al., 2019). More importantly, several ABC transporters have been frequently found to be overexpressed in a variety of tumor resistant cells, such as P-glycoprotein (P-gp, ABCB1) and breast cancer resistance protein (BCRP, ABCG2). It has been found that the ABC transporters could bind and transport chemotherapeutic drugs outside the cell by utilizing the energy provided by ATP hydrolysis, resulting in lowering the concentration of intracellular drug accumulation, reducing the efficacy of chemotherapeutic drugs and the failure of tumor treatment (Amawi et al., 2019; Liu, 2019; Eckenstaler and Benndorf, 2020). Therefore, there is an urgent need to seek small molecule inhibitors targeting ABC transporters to restore the efficacy of chemotherapeutic drugs.

A lot of studies have shown that σ_2_ receptor may be a potential therapeutic target for tumors (Zeng and Mach, 2017; Oyer et al., 2019; Schmidt and Kruse, 2019; Chen et al., 2021). The σ_2_ receptor was found to be overexpressed in rapidly proliferating tumors such as lung, breast and pancreatic cancers and was identified as an important biomarker of tumor cell proliferation (Zeng et al., 2020). And σ_2_ receptor ligands have been found to be potential agents for tumor therapy, with the capacity to inhibit proliferation and induce apoptosis in tumor cells (Nicholson et al., 2015; Cantonero et al., 2020). In addition, the σ_2_ receptor ligands exhibited promising anti-tumor proliferative activity against breast cancer MCF-7/ADR cells and could inhibit the activity of ABCB1, suggesting that σ_2_ receptor ligands may be potential therapeutic agent in anti-tumor MDR (Azzariti et al., 2006; Abate et al., 2011).

It was reported that σ_2_ receptor ligands with a 6,7-dimethoxy-1,2,3,4-tetrahydroisoquinoline structure were capable of modulating the activity of ABCB1 (Pati et al., 2018). Recently, we synthesized a series of σ_2_ ligands with 6,7-dimethoxy-1,2,3,4-tetrahydroisoquinoline structural derivatives, and found many of them showed high affinity for the σ_2_ receptor (Sun et al., 2018). Our previous study revealed that σ_2_ ligand A011 with a 6,7-dimethoxy-1,2,3,4-tetrahydroisoquinoline structure had high affinity for σ_2_ receptor and showed good antitumor activity against a variety of tumor cells, including breast cancer MCF-7, MDA-MB-231 and lung cancer A549 cells (Li et al., 2022). However, its role and mechanism in tumor resistance remains to be further investigated.

## Materials and methods

### Chemicals and reagents

The σ_2_ receptor ligand A011 was prepared as previously reported (Feng et al., 2019). KO143 was purchased from MedChemExpress (Monmouth Junction, NJ, United States). Cisplatin (DDP), adriamycin (ADR), paclitaxel, verapamil, mitoxantrone and rhodamine 123 (Rh123) were acquired from Solarbio (Beijing, China). Cell Counting Kit-8 (CCK-8) was bought from Dojindo Laboratories (Japan). Pgp-Glo™ Assay Systems were purchased from Promega (Madison, USA). Anti-P Glycoprotein antibody, ABCG2, GAPDH, Goat Anti-Rabbit IgG H&L Secondary Antibody (Alexa Fluor 488), Goat Anti-Rabbit IgG H&L Secondary Antibody and Goat Anti-Mouse IgG H&L Secondary Antibody were purchased from Abcam (Cambridge, United Kingdom). SlowFade^TM^ Gold antifade reagent was purchased from Thermo Fisher (MA, United States).

### Cell lines

ADR-resistant MCF-7 cells (MCF-7/ADR) and DDP-resistant A549 cells (A549/DDP) were purchased from Shanghai GuYan Biotech Co., Ltd. (Shanghai, China). DDP-resistant HepG2 cells (HepG2/DDP) were preserved in our laboratory. Their parental cells were originally imported from American Type Culture Collection (ATCC, Manassas, United States) and cultured with adriamycin or cisplatin to form MCF-7/ADR cells, A549/DDP and HepG2/DDP. Cells were grown in Minimum Eagle’s medium (for MCF-7/ADR), Dulbecco’s Modified Eagle Medium (for MCF-7) and RPMI 1640 (for A549, A549/DDP, HepG2, and HepG2/DDP), respectively, containing 10% fetal bovine serum (FBS), 100 U/ml penicillin, 100 μg/ml streptomycin at 37°C, 5% CO_2_. MCF-7/ADR cells were maintained using 2 µM ADR. A549/DDP and HepG2/DDP cells were maintained using 2 μg/ml cisplatin. All resistant cells were grown in drug-free medium 2 weeks before experiment.

### Cytotoxicity assay

The CCK-8 kit was used to determine the cytotoxicity of chemotherapeutic agents in MCF-7/ADR, A549/DDP, HepG2/DDP and their parental cells. In brief, 5 × 10^3^ cells per well were seeded into 96-well plates with 100 μl medium overnight. Subsequently, cells were cultured with different concentrations of A011, adriamycin, cisplatin, paclitaxel and combination, respectively. After 24, 48, and 72 h incubation, 10 μl CCK-8 was added into each well and incubated for 90 min at 37°C. The absorbance was then measured at 450 nm using a microplate reader. IC_50_ value was calculated as previously described. Survival rate = (OD_treatment_—OD_blank_)/(OD_control_—OD_blank_) × 100%, Resistant Fold (RF) = IC_50 resistant cells_/IC_50 parental cells,_ Reversal Fold (FR) = IC_50 Monotherapy_/IC_50 Combination therapy_.

### Colony formation assay

MCF-7 and MCF-7/ADR cells (5 × 10^3^ cells/well) were seeded into 6-well plates overnight. After 24 h incubation, cells were treated with A011 (0.3125, 0.625, 1.25, 2.5, 5 μM) for 24 h and cultured in drug-free medium for 10 days. During this period, cells were washed twice every other day with phosphate buffered saline (PBS) and cultured with fresh medium. Cells were then fixed with 4% paraformaldehyde for 15 min. Crystal violet was used to stain cells for 15 min. The numbers of colonies (cells >50) were counted.

### Apoptosis assay

Annexin V-FITC and propidium iodide (PI) kits were used to determine the apoptotic cells induced by A011 in MCF-7/ADR and parental cells. In short, 2 × 10^5^ cells per well were seeded into 6-well plates overnight. Different concentrations of A011 (5, 10, 20, 40 μM) was then added and incubated for 48 h. Subsequently, cells were harvested and washed twice with cold PBS before the addition of Annexin V-FITC and PI for 15 min at room temperature. Flow cytometry was used to detect the apoptotic cells.

Hoechst 33,258 fluorescent reagent was used to detect the effect of A011 on apoptosis in MCF-7/ADR and its parental cells. 2 × 10^5^ cells per well were seeded into 6-well plates. After 24 h incubation, cells were treated with A011 (5, 10, 20 μM) for 48 h. The cells were then washed with PBS and fixed, Hoechst 33,258 was added to stain for 15 min, followed by washing with PBS and photographed under the fluorescence microscope.

### Rh123 and mitoxantrone accumulation assay

Flow cytometry was used to detect the intracellular accumulation of Rh123 or mitoxantrone in MCF-7/ADR and its parental cells, with Rh123 and mitoxantrone being the fluorescent substrates for the ABCB1 and ABCG2 transporters, respectively. Briefly, 2 × 10^5^ cells were seeded into 6-well plates. After 24 h incubation, different concentration of A011 (1.25, 2.5, 5, 10 μM), verapamil (10 μM) or KO143 (10 μM) was separately added to each well and incubated for 2 h at 37°C. Subsequently, each well was washed 3 times with PBS and incubated with Rh123 (5 μg/ml) or mitoxantrone (5 μmol/L) for 2 h at 37°C. Finally, all cells were collected and resuspended with cold PBS. Flow cytometry was applied to measure the fluorescence intensity of intracellular Rh123 and mitoxantrone. Verapamil and KO143 were used as positive agent of ABCB1 and ABCG2, respectively.

### Rh123 and mitoxantrone efflux assay

Rh123 and mitoxantrone efflux were performed as previously described literature (Gao et al., 2020). In short, cells were seeded into 6-well plates (2 × 10^5^ cells per well) overnight. Cells were then incubated with Rh123 (5 μg/ml) or mitoxantrone (5 μmol/L) for 2 h at 37°C. After 2 h incubation, cells were washed 3 times with PBS and cultured with different concentration of A011 (1.25, 2.5, 5, 10 μM), verapamil (10 μM) or KO143 (10 μM) for 0, 30, 60, 90, 120 min, respectively. Subsequently, cells were collected and washed 3 times with cold PBS. Flow cytometry was used to measure the fluorescence intensity of intracellular Rh123 and mitoxantrone, respectively.

### ATPase activity of ABCB1 assay

The impact of A011 on ABCB1-mediated ATP hydrolysis was measured by the Pgp-Glo™ Assay kits. In brief, ABCB1 membranes were thawed and diluted at 4°C. Membranes were treated with various concentrations of A011 for 5 min at 37°C Mg^2+^ and ATP reagent was added into each well to trigger the reaction. After 40 min incubation, plates were removed to room temperature and ATP detection reagent was added to initiate luminescence for 20 min at room temperature. Subsequently, luminescence was read on a plate-reading luminometer.

### Immunofluorescence assay

Immunofluorescence assay was performed to detect the influence of A011 on the intracellular localization of ABCB1 and ABCG2 in MCF-7/ADR cells. In brief, 2 × 10^4^ cells per well were seed into 6-well plates overnight and treated with A011 (1.25, 2.5 μM). After 48 h incubation, cells were washed twice with cold PBS and fixed by 4% paraformaldehyde for 15 min. Bovine serum albumin (BSA) (2 mg/ml) was added to block proteins for 1 h, primary antibodies ABCB1 (1:200) or ABCG2 (1:200) were used to incubate proteins for 4 h at 4°C, which were subsequently blocked by second antibodies for 1 h. SlowFade^TM^ Gold antifade reagent was used to incubate proteins for 5 min. The fluorescence microscope was performed to collect the images.

### Western blot assay

Western blot assays were applied to detect the protein expression of ABCB1 and ABCG2 after A011 treatment in MCF-7/ADR cells. Briefly, cells were cultured with or without A011 (1.25, 2.5, 5 μM) for 48 h. Radio-immune precipitation assay (RIPA) cell lysis buffer was then used to lysis cells for 30 min on the ice. The total protein concentrations were normalized using the BCA protein assay kit. Subsequently, proteins were load and separated by SDS-PAGE kits, following transferred into polyvinylidene difluoride (PVDF) membrane and blocked by non-fat milk. Primary antibodies (ABCB1 1:1,000, ABCG2 1:1,000) were incubated for overnight at 4°C. Horseradish peroxidase (HRP)-conjugated Mouse or Rabbit antibody was used to co-incubate with PVDF membrane for 1 h at room temperature. The protein bands were visualized by Immobilon Western HRP Substrate Kits and the relative protein expression level was analysis by ImageJ software.

### Mouse xenograft assay

The MCF-7/ADR cells inoculated nude mice xenograft model was used for *in vivo* studies. The BALB/c female nude mice (4–6 weeks old, weighting 16–18 g) were bought from Guangdong Medical Laboratory Animal Centre (Guangdong, China). 1 × 10^7^ cells were injected subcutaneously into the right flank of each nude mouse. When the tumor volume reached 100 mm^3^, nude mice were randomly divided into five groups (6 mice in each group):(1) Control (saline), 2) A011 (1 mg/kg), 3) ADR (5 mg/kg), 4) A011 (5 mg/kg), 5) A011 (1 mg/kg) plus ADR (5 mg/kg) (A011 + ADR). The drugs were administered intraperitoneally every 3 days for 21 days and the body weight and tumor volume (V) of nude mice were measured according to the following formula: V = 0.5a × b^2^ [“a” is the length (mm) and “b” is the width (mm)]. The nude mice were killed by spinal dislocation and the tumor tissues were dissected and weighed. The liver, kidney and tumor samples from nude mice were fixed and preserved. All animal experiments complied with the China Animal Welfare Guide. The protocol was reviewed and approved by the Experimental Animal Research Committee of Guangdong Medical University.

### Histological analysis

The above tissue samples were dehydrated in ethanol, embedded in paraffin and sectioned with a microtome (4 μm). Sections were stained with hematoxylin and eosin for hematoxylin-eosin (HE) staining and terminal deoxynucleotidyl transferase-mediated dUTP-biotin nick end labelling (TUNEL) staining, respectively. The immunohistochemistry (IHC) staining was performed with anti-ABCG2 or anti-ABCB1 antibody. The treated samples were observed under a microscope and photographed and the images were analyzed.

### Statistical analysis

All experiments were conducted in at least three independent experiments. All data were analyzed using GraphPad Prism’s one-way ANOVA and shown as mean ± SD. Differences were considered statistically significant when the *p* value was less than 0.05.

## Results

### Cytotoxicity, resistance fold and reversal effect of A011

**FIGURE 1:**
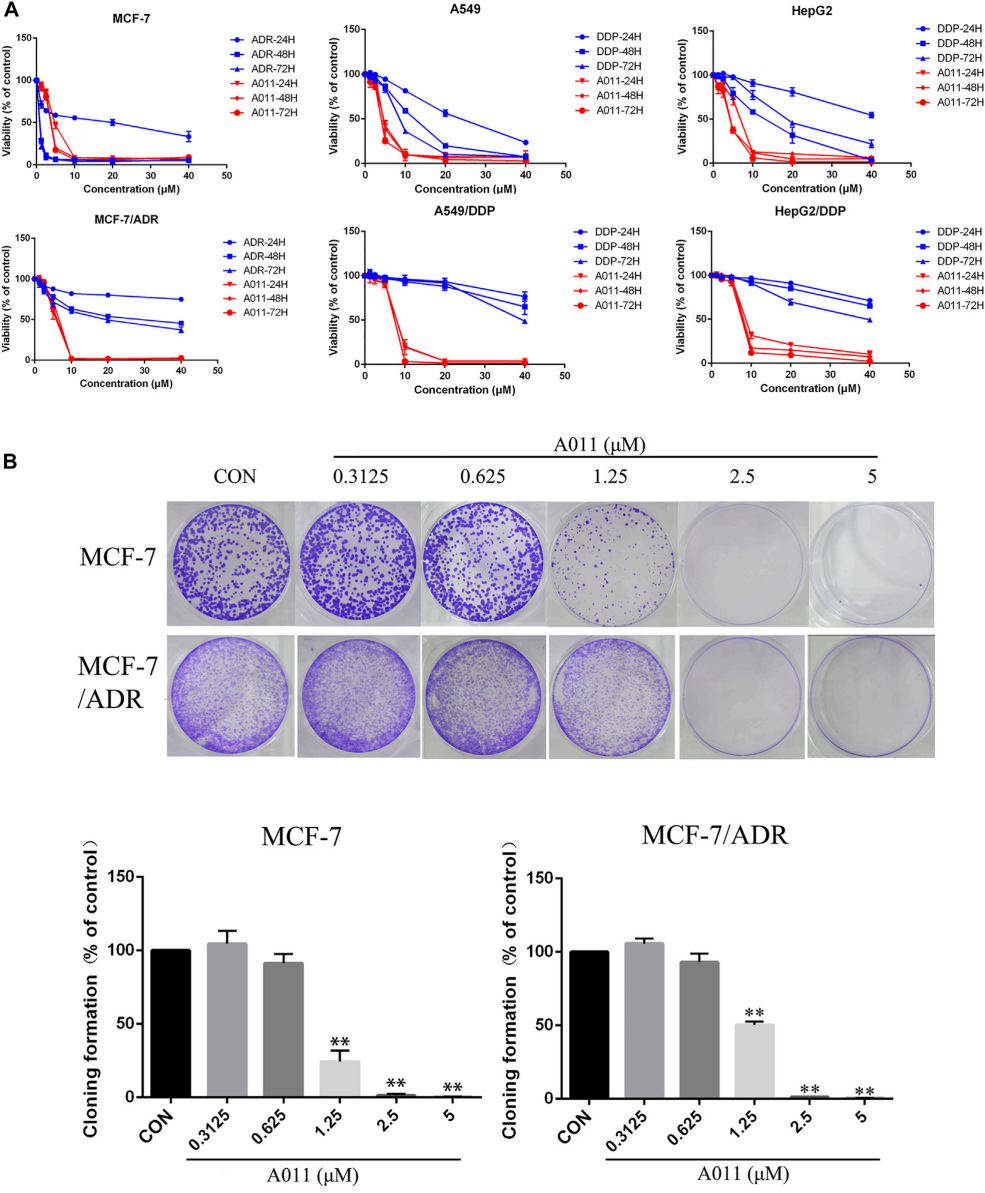
Effect of A011 on the viability of three drug-resistant cells MCF-7/ADR, A549/DDP, HepG2/DDP and their parental cells. **(A)** The cytotoxic effects of A011 on MCF-7/ADR, A549/DDP, HepG2/DDP and their parental cells, respectively. Cells were treated with a range of concentrations of A011, adriamycin (ADR) or cisplatin (DDP) for 24, 48 and 72 h. **(B)** Effect of A011 on the inhibition of clonogenic capacity of MCF-7 and MCF-7/ADR cells. Data represent mean ± SD of three different experiments. ^*^ *p* < 0.05, ^**^ *p* < 0.01 vs. Control.

We determined the cytotoxicity of A011 and positive drugs ADR, cisplatin and paclitaxel on three drug-resistant cells MCF-7/ADR, A549/DDP and HepG2/DDP and their parental cells by CCK-8 kits. ADR, cisplatin and paclitaxel showed good antitumor effects on MCF-7, A549, and HepG2 cells, with significantly decreased cytotoxic effects in MCF-7/ADR, A549/DDP, and HepG2/DDP cells, with RF value mostly >5, indicating that the 3 cell lines were multidrug resistant. In contrast, the IC_50_ values of A011 were 3.79 ± 0.13 μM, 5.35 ± 0.42 μM, RF value 1.41, 4.59 ± 0.35 μM, 7.74 ± 0.32 μM, RF value 1.69, 4.07 ± 0.25 μM, 8.29 ± 0.04 μM, RF value 2.04 for MCF-7, MCF-7/ADR, A549, A549/DDP, HepG2 and HepG2/DDP cells, respectively (Table 1). A011 showed similar anti-proliferative effects on drug-resistant cells and their parental cells, indicating that A011 had excellent anti-tumor MDR effects (Figure 1A).

**TABLE 1:** IC_50_ values and resistant folds (RF) of A011, adriamycin (ADR), cisplatin (DDP) and paclitaxel on MCF-7/ADR, A549/DDP, HepG2/DDP and their parental cells after 48 h administration.

	IC_50_(μM)		RF	IC_50_(μM)		RF	IC_50_(μM)		RF
	MCF-7	MCF-7/ADR		A549	A549/DDP		HepG2	HepG2/DDP	
A011	3.79±	5.35±	1.41	4.59±	7.74±	1.69	4.07±	8.29±	2.04
	0.13	0.42		0.35	0.32		0.25	0.04	
ADR	0.61±	27.19±	44.57	0.21±	0.20±	0.95	0.13±	48.31±	371.61
	0.02	0.28		0.01	0.06		0.12	3.10	
cisplatin	8.26±	45.83±	5.55	11.55±	72.67±	6.29	11.65±	52.90±	4.54
	0.40	1.58		0.36	2.93		0.60	0.63	
paclitaxel	0.75±	18.85±	25.13	0.42±	23.04±	54.86	0.55±	20.86±	37.92
	0.03	0.45		0.05	0.92		0.09	0.43	

Resistant Fold (RF) = IC_50_ value of drug in drug-resistant cells/IC_50_ value of drug in parental cells.

Based on the results of the CCK-8 assay, A011 had a stronger anti-proliferative effect in MCF-7/ADR cells relative to A549/DDP and HepG2/DDP cells, and therefore MCF-7/ADR cells were used as the subsequent experimental cell line. Moreover, A011 showed >90% survival of MCF-7/ADR cells at 1.25 and 2.5 μM concentrations and were therefore selected as the concentration for combination treatment. The results showed that A011 at 1.25 and 2.5 μM concentrations significantly increased the cytotoxicity of ADR, cisplatin and paclitaxel in MCF-7/ADR cells. No sensitization was observed in parental cells. And the inhibition effect of A011 combined with ADR was superior compared with that of ABCB1 inhibitor verapamil and ABCG2 inhibitor KO143 combined with ADR (*p* < 0.05) (Table 2). In addition, A011 significantly inhibited the clonogenic ability of MCF-7/ADR cells and their parental cells (Figure 1B). These results suggested that A011 has excellent anti-tumor MDR activity and could enhance the sensitivity of MCF-7/ADR cells to ADR, cisplatin and paclitaxel.

**TABLE 2:** Reversal of A011 on resistance of adriamycin (ADR), cisplatin and paclitaxel in MCF-7/ADR and MCF-7 cells.

Group	MCF-7		MCF-7/ADR	
	IC_50_(μM)	FR	IC_50_(μM)	FR
ADR	0.61 ± 0.02	1.00	27.19 ± 0.28	1.00
A011 (1.25 μM)+ADR	0.55 ± 0.08	1.11	2.89 ± 0.40	9.41^**^
A011 (2.5 μM)+ADR	0.48 ± 0.07	1.27	1.18 ± 0.26	23.04^**##^
verapamil (5 μM)+ADR	0.53 ± 0.07	1.15	4.87 ± 0.61	5.58
KO143 (5 μM)+ADR	0.51 ± 0.10	1.19	18.23 ± 1.20	1.49
cisplatin	8.26 ± 0.40	1.00	45.83 ± 1.58	1.00
A011 (1.25 μM)+cisplatin	8.38 ± 0.56	0.99	34.17 ± 2.57	1.34
A011 (2.5 μM)+cisplatin	7.57 ± 0.39	1.09	17.57 ± 1.58	2.61
verapamil (5 μM)+cisplatin	5.95 ± 0.55	1.39	21.80 ± 2.21	2.10
KO143 (5 μM)+cisplatin	7.16 ± 0.29	1.15	35.42 ± 3.86	1.29
paclitaxel	0.75 ± 0.026	1.00	18.85 ± 0.45	1.00
A011 (1.25 μM)+paclitaxel	0.70 ± 0.01	1.07	4.11 ± 0.27	4.59
A011 (2.5 μM)+paclitaxel	0.59 ± 0.02	1.27	0.91 ± 0.13	20.71^*##^
verapamil (5 μM)+paclitaxel	0.63 ± 0.01	1.19	1.79 ± 0.45	10.53
KO143 (5 μM)+paclitaxel	0.58 ± 0.03	1.29	2.21 ± 0.22	8.53

Reversal Fold (FR) = IC_50 Monotherapy_/IC_50 Combination therapy_. Verapamil as positive control group, ^*^ *p* < 0.05, ^**^ *p* < 0.01, vs. Verapamil group, ^##^ *p* < 0.01, vs. KO143 group.

### A011 induced apoptosis in MCF-7/ADR and its parental cells.

**FIGURE 2:**
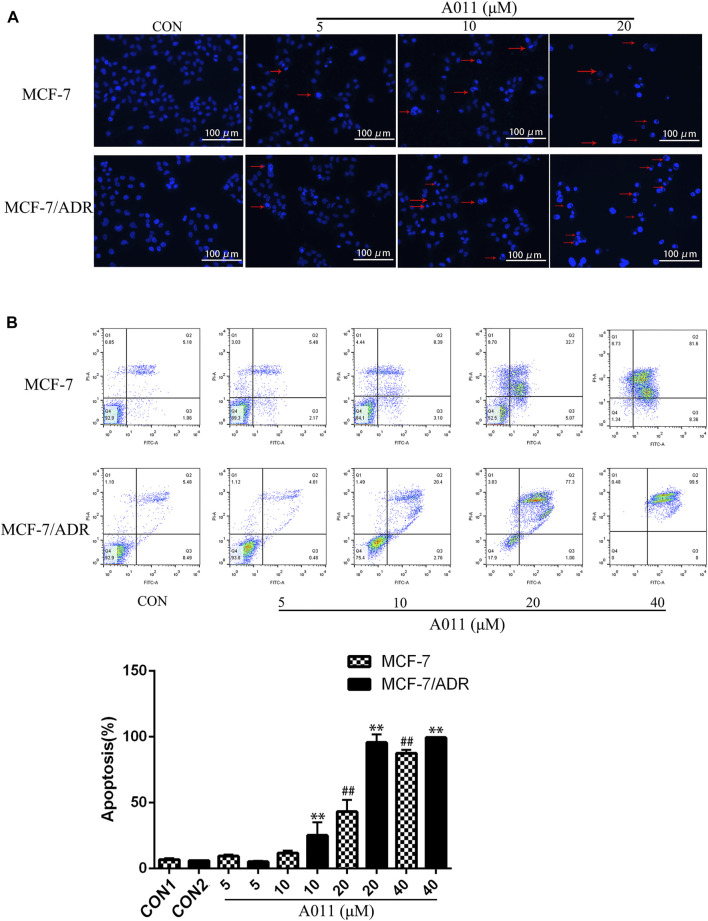
Effect of A011 on MCF-7/ADR and MCF-7 cell apoptosis. **(A)** Results of Hoechst 33,258 staining of MCF-7/ADR cells and their parental cells after A011 intervention. The red arrows indicated apoptotic vesicles. **(B)** Flow cytometry results of A011 on MCF-7/ADR cells and their parental cell apoptosis. Data represent mean ± SD of three different experiments. ##p < 0.01 vs. CON1; **p < 0.01 vs. CON2.

Hoechst 33,258 staining showed that MCF-7/ADR and MCF-7 cells exhibited increased cytoplasmic density, nuclear consolidation, nuclear membrane nucleolus fragmentation and increased apoptotic vesicles in a dose-dependent manner after A011 treatment compared to cells from control group with intact cell structure (Figure 2A). Furthermore, the flow cytometry results showed that A011 (5, 10, 20, and 40 μM) significantly induced apoptosis in MCF-7/ADR and MCF-7 cells, with apoptosis rate of 5.20% ± 0.55%, 25.15% ± 9.99%, 95.6% ± 6.35%, 99.47% ± 0.15% for MCF-7/ADR cells and 9.47% ± 1.25%, 11.87% ± 1.84%, 43.19% ± 8.81% and 87.51% ± 2.70% for MCF-7 cells (Figure 2B). The results indicated A011 could inhibit tumor cells growth by inducing cell apoptosis in MCF-7 and MCF-7/ADR.

### A011 significantly increased the accumulation and decreased the efflux of Rh123 and mitoxantrone in MCF-7/ADR cells

**FIGURE 3:**
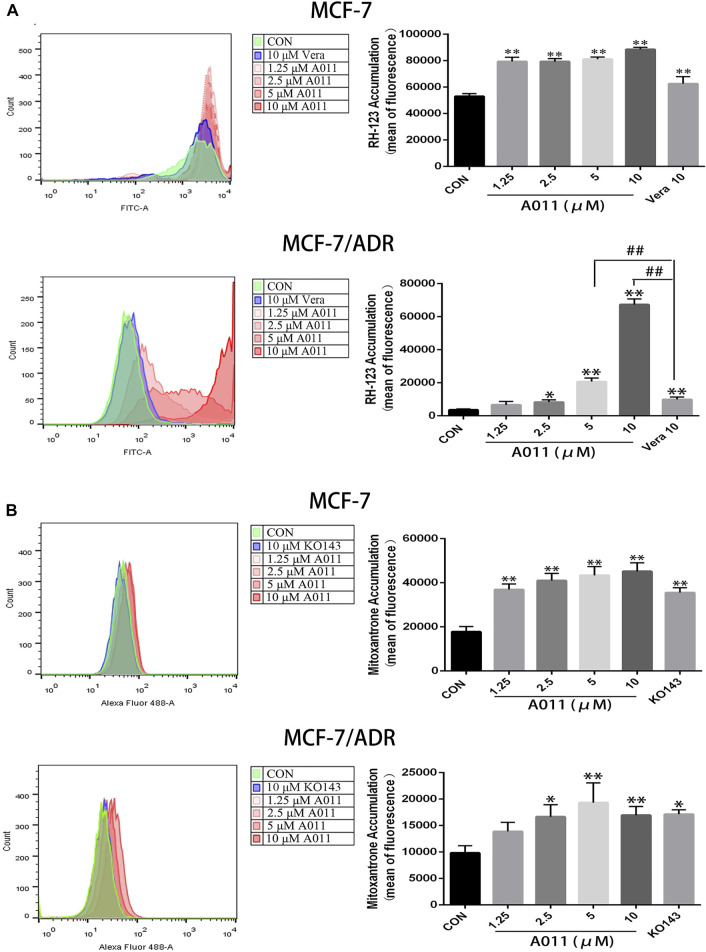
Effect of A011 on the transport function of ABCB1 and ABCG2 transporters. **(A)** A011 increased the accumulation of Rh123 in MCF-7/ADR and parental cells. **(B)** A011 increased the accumulation of mitoxantrone in MCF-7/ADR and parental cells. Verapamil (vera) and KO143 as a positive control group. Data represent mean ± SD of three different experiments. *p < 0.05, **p < 0.01 vs. CON.

**FIGURE 4:**
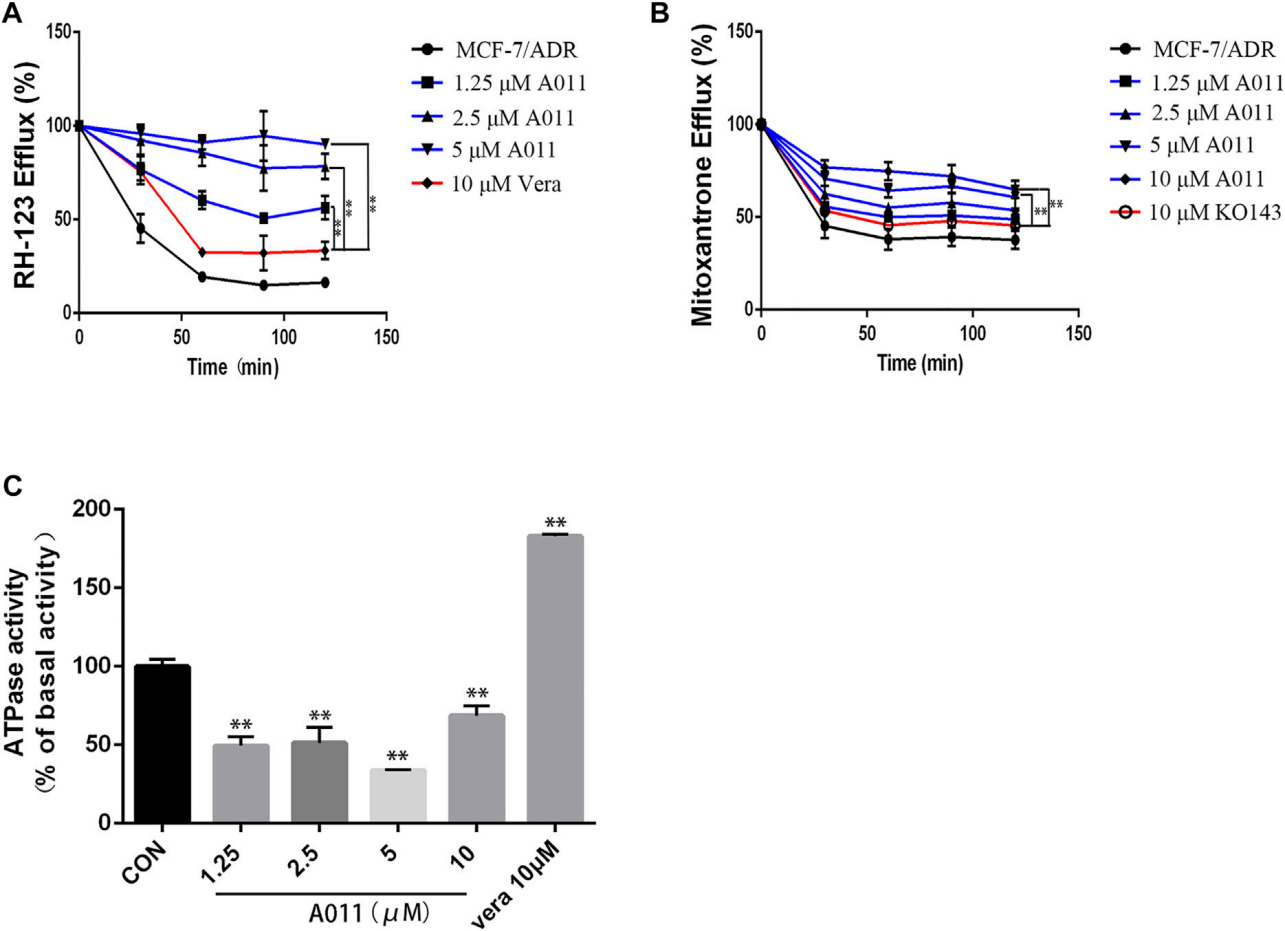
Effect of A011 on the transport function of ABCB1 and ABGC2 transporters. **(A)** A011 decreased the efflux of Rh123 in MCF-7/ADR cells. **(B)** A011 decreased the efflux of mitoxantrone in MCF-7/ADR cells. **(C)** A011 decreased the ATPase activity of the ABCB1 transporter. Verapamil (vera) and KO143 as a positive control group. Data represent mean ± SD of three different experiments. **p < 0.01 vs. CON.

To investigate the mechanism by which A011 reversed drug resistance in tumor cells, we determined the effect of A011 on the function of ABCB1 and ABCG2 transporters. Verapamil and KO143 were inhibitors of the ABCB1 and ABCG2 transporters respectively, and were able to inhibit the transport of substrates outside the cell membrane by the ABCB1 and ABCG2 transporters, so they were used as positive control. In addition, Rh123 and mitoxantrone were fluorescent substrates for the ABCB1 and ABCG2 transporters respectively, and were able to be quantified by flow cytometry. The results showed that the accumulation of Rh123 or mitoxantrone in MCF-7/ADR cells was significantly lower than their accumulation in parental cells. A011 significantly increased the accumulation of Rh123 and mitoxantrone in MCF-7/ADR cells. Moreover, the intracellular accumulation of Rh123 was higher in the A011 group compared to the verapamil group (Figures 3A,B). In addition, efflux experiments showed that the amount of Rh123 or mitoxantrone in MCF-7/ADR cells was significantly reduced during the time course, whereas the addition of A011 significantly inhibited the efflux of Rh123 or mitoxantrone, suggesting that the increased accumulation of Rh123 and mitoxantrone in MCF-7/ADR cells was due to inhibition of their efflux (Figures 4A,B). These results indicated that A011 could inhibit the transport function of ABCB1 and ABCG2 transporters.

### A011 significantly decreased the ATPase activity of the ABCB1 transporter

To further investigate the role of A011 on the function of the ABCB1 transporter, we measured the effect of A011 on the ATPase activity of the ABCB1 transporter. The results showed that verapamil increased the activity of ABCB1 transporter ATPase, which was in agreement with the description of previous studies (Ledwitch et al., 2016). In contrast, A011 could significantly decrease the activity of ABCB1 transporter ATPase compared to the control group, indicating that A011 could inhibit the transport function of ABCB1 transporter by inhibiting the activity of ABCB1 transporter ATPase (Figure 4C).

### A011 Decreased ABCG2 Protein Expression without Altering ABCB1 Protein Expression and Localization of ABCG2 and ABCB1 Proteins in MCF-7/ADR Cells

**FIGURE 5:**
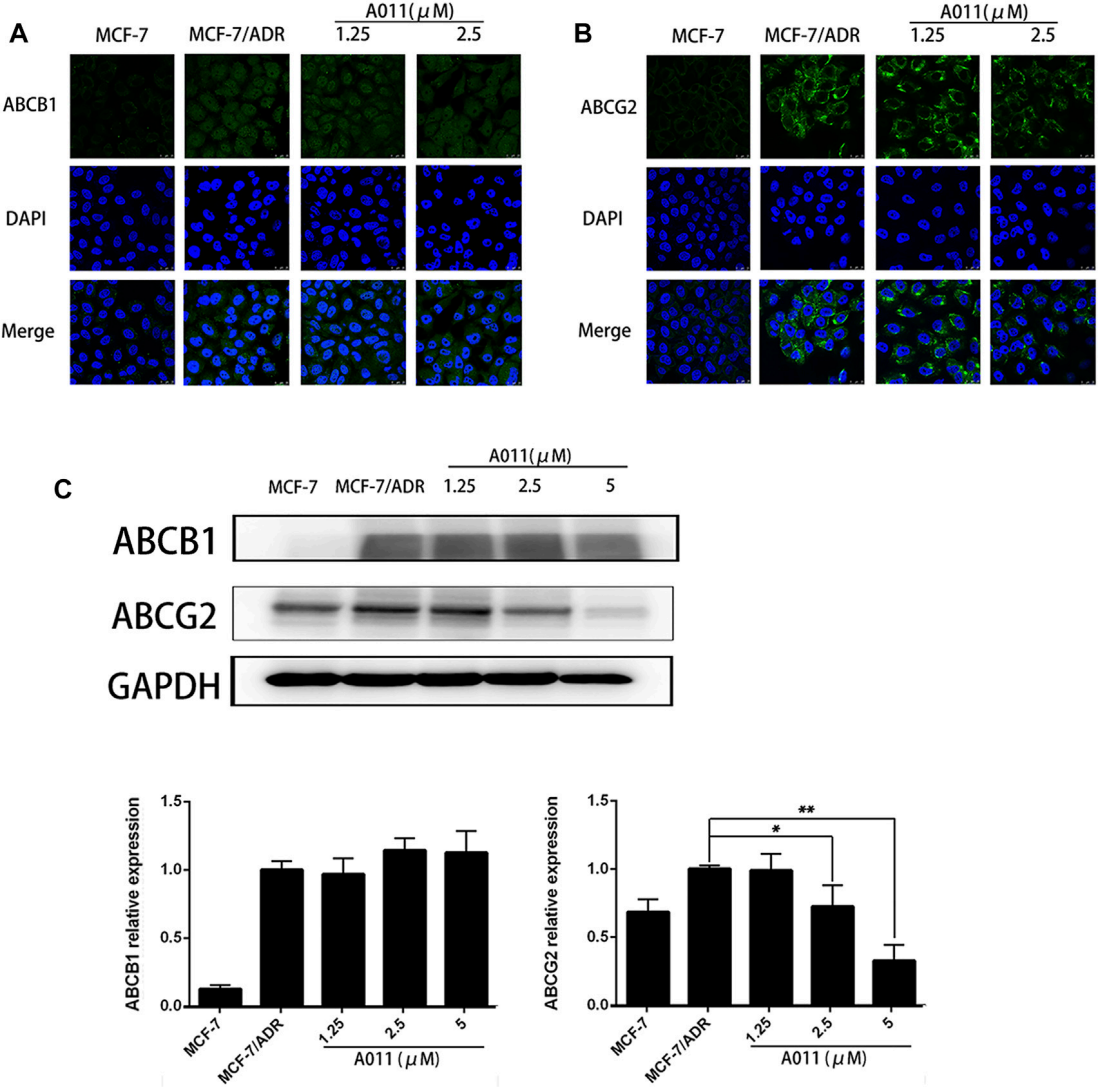
Effect of A011 on the expression and intracellular localization of ABCB1 and ABCG2 proteins. **(A)** A011 did not alter the localization of ABCB1 protein in MCF-7/ADR cells. Cells were treated with A011 for 48 h and then detected by immunofluorescence assay. **(B)** A011 did not alter the localization of ABCG2 protein in MCF-7/ADR cells. **(C)** A011 decreased ABCG2 protein expression in MCF-7/ADR cells without altering ABCB1 protein expression. Cells were treated with A011 for 48 h and then detected by western blot assay. Data represent mean ± SD of three different experiments. *p < 0.05, **p < 0.01 vs. Control. A011 Inhibited the Growth of MCF-7/ADR Xenograft Model in vivo.

Given reducing the expression of transporter proteins and altering their localization in cells is one of the mechanisms to overcome MDR, we further investigated the effect of A011 on ABCB1 and ABCG2 proteins in MCF-7/ADR cells by immunofluorescence and western blot. Immunofluorescence assay showed that A011 did not alter the localization of ABCB1 and ABCG2 proteins in MCF-7/ADR cells (Figures 5A,B). And western blot showed that A011 down-regulated ABCG2 protein expression and did not alter ABCB1 protein expression (Figure 5C).

**FIGURE 6:**
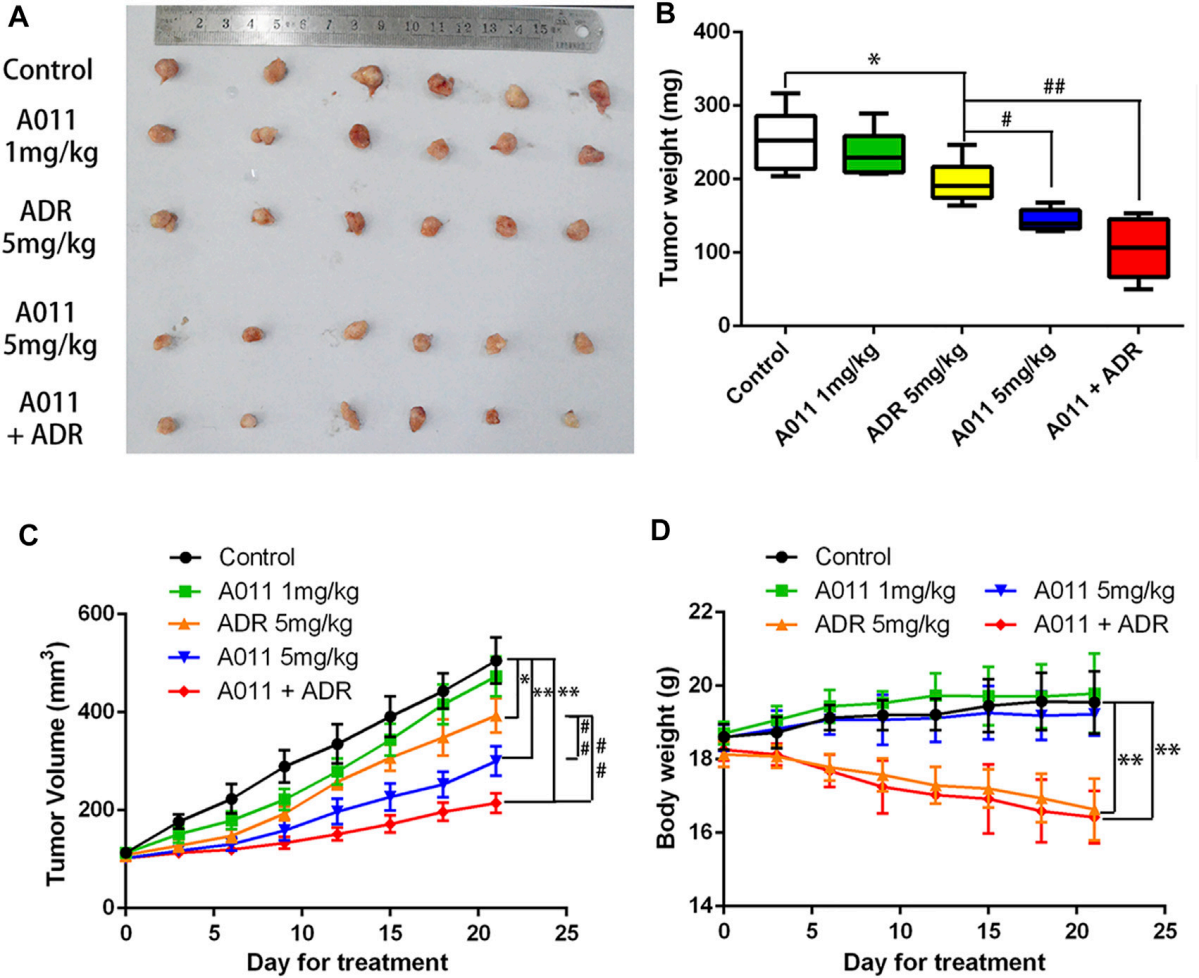
A011 inhibited the growth of MCF-7/ADR xenograft model in vivo. MCF-7/ADR cells were injected subcutaneously into the right flank of BALB/c-nu/nu nude mice. **(A)** Photographs of tumors. **(B)** Mean tumor weight was calculated for each group of tumors after dissection of nude mice. **(C)** Change in tumor volume over the 21 days treatment period. **(D)** Change in body weight of nude mice during the 21-days treatment period. Nude mice in Control group treated with saline. Nude mice in the A011 + ADR group were treated with A011 (1 mg/kg) in combination with ADR (adriamycin) (5 mg/kg), while the other groups were treated as depicted above. ADR: adriamycin. Data represent mean ± SD of three different experiments. *p < 0.05, **p < 0.01 vs. Control. #p < 0.05, ##p < 0.01 vs. ADR (5 mg/kg). A011 Induced Apoptosis and Downregulated ABCG2 Protein Expression in MCF-7/ADR Tumor Cells without Significant Toxicity to Liver and Kidney

To evaluate the anti-tumor MDR activity of A011 *in vivo*, we established a xenograft model with MCF-7/ADR cells in nude mice. A011 (1 mg/kg) or ADR (5 mg/kg) alone showed low growth inhibitory activity against MCF-7/ADR tumors, with growth inhibition rates of 6.94% and 22.58%, respectively. However, when ADR (5 mg/kg) was co-administered with A011 (1 mg/kg), the anti-tumor activity of ADR was significantly enhanced with the growth inhibition rate of 58.43%. A011 (5 mg/kg) alone showed good anti-tumor activity against MCF-7/ADR tumors with the growth inhibition rate of 43.13% (Figures 6A–C). In addition, there was no significant change in body weight in the A011 group compared to the saline group (Figure 6D). These results suggested that A011 alone or in combination had promising anti-tumor MDR activity and was well tolerated *in vivo*.

**FIGURE 7:**
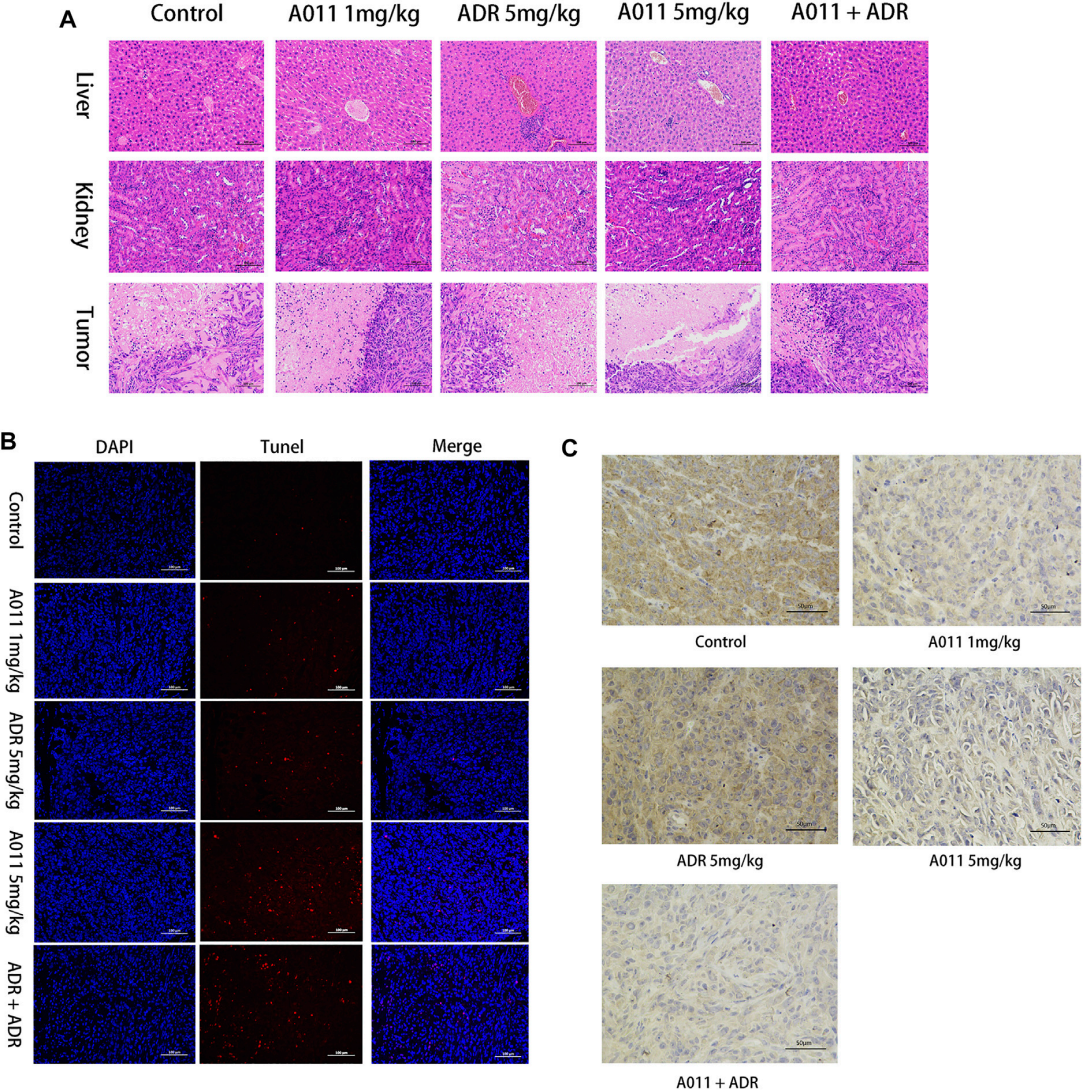
Histological analysis results. **(A)** HE staining of liver, kidney and tumor tissue (200×). **(B)** TUNEL staining of each group of tumor tissue. **(C)** Immunohistochemistry of ABCG2 protein in each group of tumor tissues. ADR: adriamycin. Scale bar = 100 μm or 50 μm.

To evaluate the effects of A011 in liver, kidney and tumor tissues in nude mice, we performed HE tissue staining, TUNEL staining and immunohistochemistry experiments respectively. HE staining of the liver and kidney showed that there was no difference between all treatment groups and control group, except for a small amount of inflammatory cell infiltration in the ADR (5 mg/kg) and A011 combined with ADR groups. In HE staining of tumor tissue, approximately 60% and 70% of tumor cells were necrotic in A011 (5 mg/kg) and A011 combined with ADR respectively, compared to 40% in the control group (Figure 7A). TUNEL staining showed that administration of A011 (5 mg/kg) alone or A011 (1 mg/kg) in combination with ADR (5 mg/kg) significantly induced an increase in MCF-7/ADR tumor apoptosis (Figure 7B). Immunohistochemistry of ABCG2 showed dark brown staining of ABCG2 protein in the saline and ADR (5 mg/kg) groups. As the concentration of A011 increased, the expression of ABCG2 was down-regulated (Figure 7C). A011 did not alter the expression of ABCB1 (Supplementary Figure S1). Above results suggest that A011 could induce apoptosis and down-regulate ABCG2 protein expression in MCF-/ADR tumor cells *in vivo*, without significant toxicity to liver and kidney tissues.

## Discussion

Intrinsic MDR and acquired MDR are two of the major barriers to tumor treatment, seriously threatening the survival and affecting the lives of cancer patients (Zheng, 2017; Al-Akra et al., 2019). Due to the narrow therapeutic window of most chemotherapeutic agents, the emergence of tumor MDR has greatly limited the clinical use of chemotherapeutic agents, therefore it is particularly important for cancer treatment to overcome tumor MDR.

The σ_2_ receptor has been found to be highly expressed in rapidly proliferating tumors such as breast cancer and is considered to be one of the potential targets for the treatment of tumors (Huang et al., 2014). Most of the σ_2_ ligands not only show high affinity for the σ_2_ receptor but also exhibit excellent anti-tumor activity (Georgiadis et al., 2017; Sun et al., 2018; Liu et al., 2019), and those with a 6,7-dimethoxy-1,2,3,4-tetrahydroisoquinoline structure could also interact with ABCB1 (Pati et al., 2015). Moreover, a few σ_2_ receptor agonists were found to be collateral sensitive and their anti-proliferative activity was stronger in cells with high P-gp expression than in P-gp negative cells, suggesting that σ_2_ receptor ligands may have promising activity in drug-resistant tumors (Niso et al., 2013; Abatematteo et al., 2021). In this study, we found that σ_2_ ligand A011 showed significant cytotoxic activity in three tumor cell lines MCF-7, A549 and HepG2 cells and generally showed no decrease in cytotoxic activity in drug-resistant cell lines MCF-7/ADR, A549/DDP and HepG2/DDP cells. In addition, A011 significantly increased the sensitivity of MCF-7/ADR cells to ADR at concentrations of 1.25 and 2.5 μM. And *in vivo* experiments, A011 (5 mg/kg) alone or A011 (1 mg/kg) co-administered with ADR showed promising anti-tumor activity, significantly inhibiting the growth of MCF-7/ADR tumors without significant toxicity, suggesting that A011 has the potential to overcome MDR.

Apoptosis is a form of programmed cell death that is co-regulated by multiple genes with important roles in maintaining the homeostasis of the internal environment and controlling cell proliferation (Goldar et al., 2015)_._ Dysregulation of apoptosis is one of the hallmarks of cancer and is associated not only with tumorigenesis and progression, but also with tumor resistance to chemotherapeutic agents (Pistritto et al., 2016). We found that A011 could dose-dependently induce apoptosis in MCF-7/ADR and its parental cells, and the apoptosis induction of A011 was better in MCF-7/ADR than MCF-7 cells. In a MCF-7/ADR xenograft model, A011 (5 mg/kg) was able to induce an increased apoptosis relative to the control group, suggesting that A011 may also promote cell death by inducing MCF-7/ADR apoptosis.

ABC transporters have been found to be relatively highly expressed in drug-resistant tumors and able to transport intracellular chemotherapeutic agents to the extracellular compartment by relying on the energy provided by ATP hydrolysis, thereby mediating the resistance of tumor cells to chemotherapeutic agents (Choi and Yu, 2014; Beis, 2015). To elucidate the mechanism that A011 overcame MDR in MCF-7/ADR cells, we examined the activity and protein expression of the ABCB1 and ABCG2 transporters. Our results showed that A011 could inhibit ABCB1 transport function and ATPase activity, but had no effect on its protein expression. In addition, it was first discovered that A011 also inhibited the transport function and protein expression of ABCG2, which was further validated in ABCG2 protein immunohistochemical assay *in vivo*. Besides inhibiting ABC transporter activity and protein expression, ABC transporters as transmembrane proteins, altering their localization in cells is also part of the strategy to inhibit ABC transporter-mediated MDR (Zhao et al., 2019). However, A011 did not affect the localization of the ABCB1 and ABCG2 transporters in MCF-7/ADR cells. These results suggested that A011 could inhibit the function of ABCB1 and ABCG2 transporters and reduce the expression of ABCG2 protein, thereby overcoming MDR. However, the mechanisms of A011 inhibiting ATPase activity and expression of ABCG2 protein remain to be clarified.

A variety of σ_2_ ligands are currently being developed for clinical diagnosis and cancer treatment with PET imaging of tumors. Phase I clinical trial results for the σ_2_ radioligand [^18^F]ISO-1 showed that [^18^F]ISO-1 uptake values correlated with tumor Ki-67 (a gold standard proliferation biomarker) and are expected to be used for *in vivo* measurement of tumor proliferation status (Dehdashti et al., 2013). Studies have shown that a lot of selective σ_2_ receptor ligands displayed cytotoxic effects on a variety of human cancer cells, and inhibited tumor growth (Asong et al., 2019; Liu et al., 2019). We previously found that A011 was able to increase intracellular ROS and Ca^2+^ levels in MCF-7 cells and induced endoplasmic reticulum stress and autophagy (Li et al., 2022). In addition, the study showed that A011 was well tolerated and had no significant toxicity to liver and kidney tissues. These results provided further insight into the pharmacological role of the σ_2_ receptor and A011 may be a candidate for cancer treatment either alone or in combination with other anticancer agents.

## Conclusion

In this study, we elucidated that the σ_2_ ligand A011, containing a 6,7-dimethoxy-1,2,3,4-tetrahydroisoquinoline structure, exhibited excellent anti-breast cancer MDR activity both *in vivo* and *in vitro*, either alone or in combination with ADR. A011 demonstrated anti-MDR activity by inhibiting the transporting function of ABCB1 and ABCG2 transporters and thus was a potential therapeutic agent for the treatment of tumor resistance.

## Data Availability

The original contributions presented in the study are included in the article/Supplementary Materials, further inquiries can be directed to the corresponding authors.
