# Supplementary figures and images for: Novel Sigma-2 receptor ligand A011 overcomes MDR in adriamycin-resistant human breast cancer cells by modulating ABCB1 and ABCG2 transporter function
Source: Front Pharmacol. 2022 Aug 31;13:952980. doi: 10.3389/fphar.2022.952980 (PMC9473340; doi:10.3389/fphar.2022.952980)

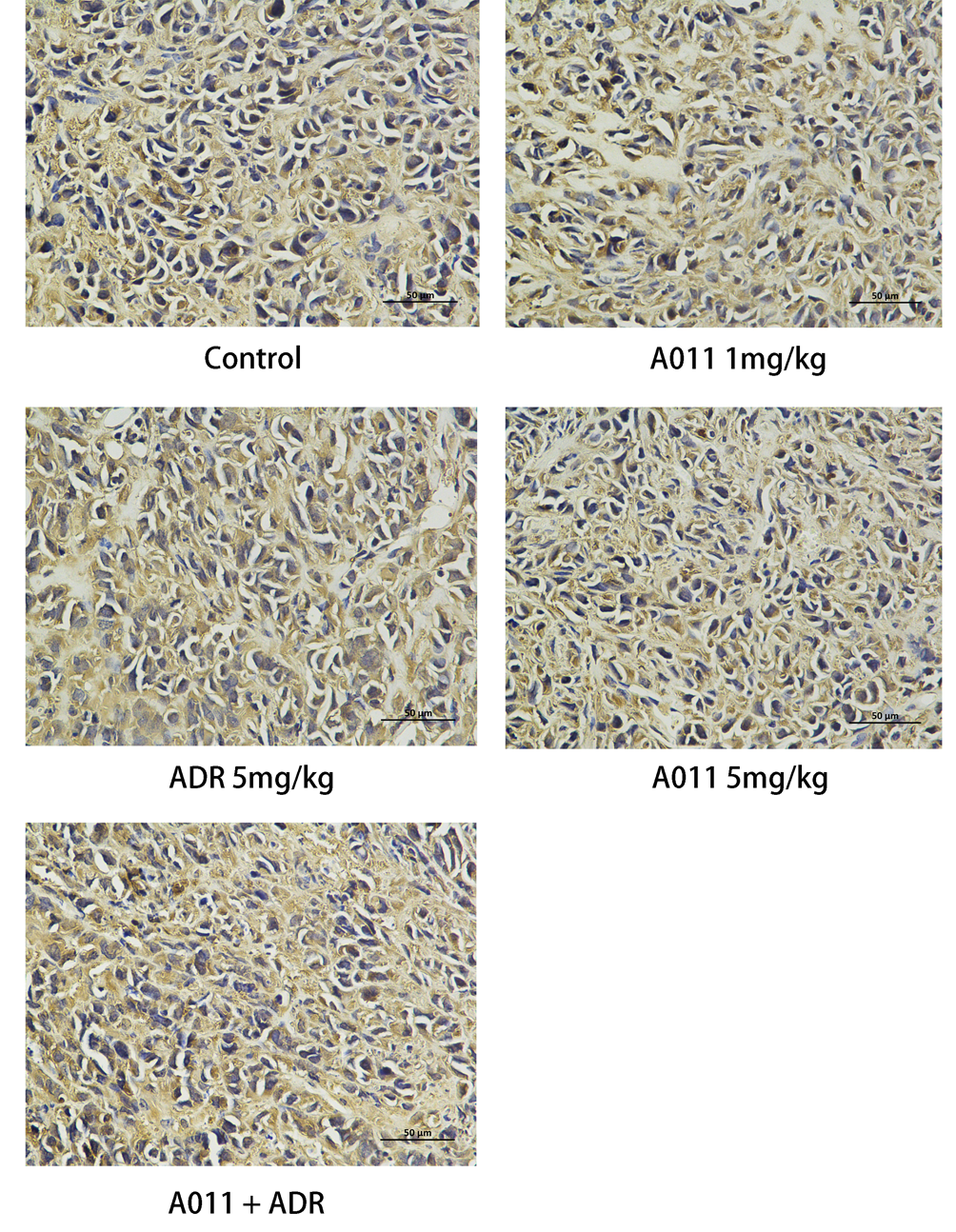

Supplement: Supplementary file 1 [file Image1.tif]
